# Hospital Sunday Fund

**Published:** 1896-02-08

**Authors:** 


### The Institutional Workshop.

### HOSPITAL SUNDAY FUND.

An important meeting of the Council of the Metropolitan Hospital Sunday Fund was held on Thursday last, January 30th, at the Mansion House, under the presidency (in the unavoidable absence of the Lord Mayor) of the Bishop of Stepney (Dr. Browne). Among those present were the Earl of Stamford, the Dean of St. Paul's, Archdeacon Sinclair, the Very Rev. Canon Graham (representing Cardinal Vaughan), Rev. Canon McCormick, Rev. Prebendary Kitto, Rev. W. H. Barlow, Rev. M. R. Neligan, Rev. H. Russell Wakefield, Rev. Dr. Kennedy, Rev. Dr. Rigg, Rev. S. B. Burnaby, Rev. W. T. Du Boulay, Rev. C. H. Grundy, Rev. A. A. Harland, Rev. W. H. Harwood, Sir Savile Crossley, Mr. Reginald Acland, Mr. Thomas Bryant, P.R.C.S., Mr. Henry C. Burdett, Mr. Thomas Christy, Mr. Alfred L. Cohen, Dr. J. G. Glover, Mr. J. H. Hall, Mr. John Mackrell, Mr. C. Macnamara, F.R.C.S., Captain A. Palliser, Mr. Howard Potter, Mr. W. R. Tidd, Captain Cundy, Mr. F. H. Norman, Dr. Sedgwick Saunders, F.S.A., Mr. Alfred Willett, F.R.C.S., and Mr. H. N. Custance (secretary).

Before proceeding with the business of the day, it was unanimously resolved, on the motion of the Chairman, that the Council desired to convey to Her Majesty the Queen and the Princess Beatrice an expression of their earnest and deep sympathy in their loss and bereavement occasioned by the death of H.R.H. Prince Henry of Battenberg.

The following were reappointed as the Committee of Distribution for the current year, viz., the Lord Mayor, Sir Sydney H. Waterlow (vice-president), Sir Savile Crossley, Alderman Sir David Evans, K.C.M.G., Mr. Bonsor, M.P., Captain J. Cundy, Mr. Herman Hoskier, Mr. F. H. Norman, Dr. Sedgwick Saunders, and Mr. Alfred Willett. The General Purposes Committee were also reappointed. Sir Edmund Hay Currie and Mr. Richard B. Martin, M.P., were re-elected honorary secretaries, which position they have held since the inauguration of the movement, and Mr. Henry N. Custance was reappointed secretary. Messrs. W. H. Pannell and Co., of Basinghall Street, were selected, for a second year, to be honorary auditors of the fund.

### The Out-patient Question.

Dr. Glover then moved that a committee be appointed to investigate and report on the out-patient system of the hospitals. He said they all regretted the absence of Sir Sydney Waterlow on this occasion, and they would be anxious to have his opinion on the subject. He (Dr. Glover) might remind them that notice was given of this subject last year, when Sir Sydney Waterlow was himself in the chair. He thought that there was a general concurrence of opinion in the Council that the subject was a reasonable one for their discussion. Dr. Glover read a letter from the Rev. Mr. King, Vicar of Sydenham, calling attention to the abuses of the out-patient system, which abuses consistedin promiscuous crowding and consequent danger of infection, the misuse of the system by the middle classes and servants in situations, the overlapping of cases and need of registration, the brevity of the medical examination of the patients and the long hours which such patients had to wait. Dr. Glover said said he might remind them that there was a very important inquiry into the subject in 1871, presided over by the late Sir W. Fergusson in London, and there was also one in Birmingham. The Lords' Committee which sat two or three years ago took a great deal of evidence and made some important remarks on the matter. Mr. Burdett, in the course of his great work on hospitals, had entered fully into the subject, which was one that was constantly cropping up before the public, and was one of great moment to the welfare and prosperity of the London hospitals. As representing the charitable and religious public, it was their duty to look at this from a charitable, religious, and public point of view. In entering upon a discussion of this kind they did so with the most friendly feelings for the hospital system. Last year no fewer than 1,052,932 out-patients were treated in the London hospitals, exclusive of the great endowed hospitals of St. Thomas's and St. Bartholomew's, and over 250,000 more in the various dispensaries. The expenditure on the out-patient system was £134,690, or about 3s. 5d. per head. The system, which was considered an excrescence on hospital work, increased year by year. There was even an excrescence on an excrescence, and that was the casualty system—people dropped into hospitals without letters of recommendation just casually, and were seen once or twice, though in the great majority of cases he believed they were only seen once. At Guy's Hospital the total number of out-patients in 1891 was 57,848, and three years later the number had risen to 88,753. There were, in addition, at all the hospitals casualty cases involving instant treatment for temporary and small accidents. At St. Bartholomew's these casualties amounted sometimes to 390 a day. It was their duty as friends of the hospitals, and as supporters of the hospital system, to inquire into the matter. The out-patient system in London was not justified even by the medical men who administered it. He had often seen medical men exhausted after a few hours in the out-patient department. It was not essential to the hospital system. There had been out-patients from the beginning, and he thought there would have to be out-patients to the end, but it was not an essential part of the system. It was not fair to the medical men, and it was not fair to the poor, who were often detained for hours while suffering from cancer, consumption, and other grave diseases. The practice was not that which made the fame of hospitals. It was said to be good for medical students, but he denied that that was the way to teach them. The system had a very bad reflex action on the medical profession. Practitioners were put in competition with it; they were driven to accept small fees, and were compelled to attend patients on terms which were absolutely inconsistent with the proper treatment of the case. He did not desire the extinction of the system, or to do anything to prejudice the medical treatment of the deserving poor, but he considered that he had made out a case for an inquiry into the system, with a view, if possible, to its curtailment.

The Rev. Prebendary Kitto seconded the motion, observing that he did so not because he agreed with all that had been said, but because he thought a case for inquiry had been made out. He desired that the facts should be ascertained, and that a report should be made to the Council of any remedies which it was proposed to adopt. He was not sanguine as to the result, except that it would inform the public mind.

Canon Graham spoke in opposition to the motion. There was undoubtedly great crowding, only a brief examination of the patients, and long hours of waiting, and these, no doubt, were great evils; but if a committee were appointed he did not think they would know more than they did already.

The Rev. C. H. Grundy also opposed the appointment of a committee.

Mr. Alfred Cohen supported the motion. There was no doubt that there were abuses in the out-patient system difficult to remedy. A great many of those abuses had been remedied in past years, and probably if it was known by each individual hospital what other hospitals had done—some of them successfully—to remedy them it would have a good effect. Probably a vast amount could be done with the friendly co-operation of the hospitals themselves. He suggested that the larger hospitals should be represented on the proposed committee. He did not want for a moment to interfere with the management of the hospitals. He thought that Dr. Glover's committee, with the aid of the representatives of the large hospitals, could produce nothing but good results.

The Rev. Dr. KENNEDY questioned the right of the Council to interfere materially with the internal management of hospitals. They would be entering upon a rather dangerous course of proceeding if they made it plain to the public that they claimed a right to go inside the doors of hospitals, and to ascertain for themselves whether everything there was done in the best possible way. That there were abuses in the present out-patient system he supposed they must all admit. But if he understood Dr. Glover, what he desired was the abolition of the out-patient system.

Dr. GLOVER: No; I said I did not wish that.

The Rev. Dr. KENNEDY, continuing, referred to the benefits which the poor derived from the out-patient system. Whatever defects there might be in that system there must be something of the same kind provided for the poor of London. Out-patient letters were valued extremely. He did not think that the hospitals would have the right to shut up the out-patient department. He had known most distressing cases in London where persons, instead of going to hospitals, employed a medical man, and consequently incurred debts, the medical man taking a payment of 1s. a week from these people, who might have gone to the hospital and obtained the treatment for nothing.

The Rev. Dr. RIGG thought that the resolution might be expressed in terms which would not lay it open to any misconception. He agreed with the Rev. Dr. Kennedy that they had no right to attempt to interfere with the control of hospitals, but he did not think that that was contained in the resolution. He did not think that the resolution went beyond seeking information and offering suggestions. He considered that the proposed inquiry would do a great deal of good. Perhaps it would meet the case if the Committee of Distribution were to make the inquiry. He suggested that it should be an instruction to the Distribution Committee to inquire into the facts and to obtain suggestions, and for that purpose the committee might associate with itself any members of the Council whose co-operation it might desire. He considered that that would meet the case. He regarded Mr. Cohen's suggestion that hospitals should be represented as a valuable one. He moved as an amendment, "That it be an instruction to the Committee of Distribution to more fully consider the subject of hospital out-patient departments, and that they have power to invite the co-operation of other members of the Council."

The Rev. Dr. KENNEDY seconded the amendment.

Mr. BURDETT said that the managers of hospitals were as anxious as anyone to reduce the number of out-patients, but they felt that they were not in a position to do this because the means of reduction rested rather with the medical profession than with the lay managers of hospitals. At the present time all the medical men gave their services free to the hospitals, and the governing bodies felt that if the medical profession desired, as they did desire, to maintain an increase in the numbers of the out-patients admitted, they (the managers) did not see—and experience proved that they were justified in their view—how it was possible for them, in face of the doctors' attitude on the question, to reduce the number of out-patients. The restriction of the number of out-patients should be enforced in a businesslike way. Dr. Glover was one of the Members of Parliament for the medical profession, as he had been elected by the great body of general practitioners as their direct representative on the Medical Council. If the general body of medical practitioners, whose practices were being ruined by the present system of out-patient relief, chose to co-operate together and make urgent representations to the members of the honorary medical staff of each of the great hospitals, the attitude of the members of each medical staff must alter, and then, but not till then, would the out-patient system cease to be a byeword. Then, but not otherwise, it would be managed with some regard to the interests of the great body of the medical profession and of the public themselves. He thought that Dr. Rigg's suggestion was a wise one. If the matter was referred to the Distribution Committee it would be considered very carefully. He thought that a little delay in deciding what form the inquiry should take would be profitable, and a benefit in every way. The Distribution Committee would consider what form the inquiry should take, and would bring up a report to the Council.

The CHAIRMAN, in the course of the discussion, read the following extract from a letter written by Sir Sydney Waterlow: "I understand that Dr. Glover has given notice of his intention to move a resolution in reference to the out-patient system at the several hospitals. There can, of course, be no objection to a committee, if the meeting are of opinion that any further information will be obtained by adopting such a course beyond that which is every year supplied to the Distribution Committee. It should be borne in mind that this committee, when considering the bases of award to be recommended for each hospital, dispensary, &c., have before them the number of out and lying-in patients and the total number of attendances. They have also a return of the cost of each out and each in-patient, and, as far as possible, they endeavour to ascertain what kind of supervision is exercised over the out-patient department, with a view to prevent persons obtaining gratuitous medical relief who can afford to pay a doctor. These circumstances and the statistics referred to are very carefully weighed before determining the award to be recommended. The management of the out-patient departments in the several hospitals where very large numbers are treated is not, perhaps, as perfect and complete as the public have a right to expect, although much improved of late years. If the meeting express an opinion to that effect, the Distribution Committee would naturally act more stringently whenever they found that proper supervision had not been exercised over the out-patients."

Dr. GLOVER said he had no wish to close the out-patient department. He accepted the amendment of the Rev. Dr. Rigg, but he did not do so with entirely personal concurrence, as he thought that the Distribution Committee should be reserved for distribution purposes. As the opinion of the meeting was that it should be referred to that committee, he accepted the amendment.

The amendment moved by the Rev. Dr. Rigg was then agreed to.

### THE METHOD OF DISTRIBUTION.

Mr. R. B. D. ACLAND moved: "That a committee of six members of the Council, the president, vice-president, and the hon. secretaries *ex officio*, be elected to consider and report whether grants could be made under the present system to Guy's and St. Thomas's Hospitals adequate to their needs, and, if not, whether any and if so what alteration should be made in the laws of the constitution." He spoke at length in support of the resolution, and pointed out that Guy's Hospital was the second largest hospital in London and St. Thomas's Hospital the third largest. Guy's Hospital appeared in the list of awards as 18 and St. Thomas's did not appear at all. They were told that they were bound by the laws of the constitution. The 5th law was the one bearing on the subject, and it was as follows: "The awards to hospitals, &c., shall be primarily based on the average total expenditure of each institution for the last three years, after deducting therefrom (1) a sum equal to the income derived from endowments and realised property; (2) the amount received in legacies exceeding £100 each, unless such legacies have been necessarily spent to meet the current expenditure of the institution; (3) The amount of expenses of management; but in every case the merits and pecuniary needs of the institution concerned shall be fully inquired into, and considered by the Distribution Committee, and the award made shall be determined in accordance with the judgment of the Distribution Committee upon such merits and needs, provided that in no case shall the grant be further reduced or withheld until a conference shall have been sought with the managing committee of the said hospital, &c." If it be that that rule prevented adequate grants being made to Guy's Hospital and St. Thomas's Hospital, then it should be abolished.

Dr. Sedgwick Saunders pointed out that St. Thomas's Hospital had never asked for a grant.

Mr. Norman seconded the resolution.

Mr. Burdett pointed out that the rule as it at present operated did not prevent adequate grants from being made to any hospital. Whatever method of distribution was adopted it would, no doubt, be open to objection on some points. The Distribution Committee of the Fund commanded the confidence not only of the hospitals as a whole, but of the public, a fact which the result of last year's collections proved to demonstration. Mr. Acland had spoken of the case of Guy's, the London, St. George's, Charing Cross, and St. Thomas's Hospitals. The case of St. Thomas's was exceptional from the circumstance that it received in 1894 an available income of £44,870 from investments and patients' payments, and that its expenditure amounted to only £44,259. A comparison between Guy's and the London Hospitals would readily account for the differences in the grants made to these and the other institutions. In 1894 Guy's had an assured income of £27,686, which was made up as follows: Invested property, £22,398; probationers' fees, £902; patients' payments, £4,386; its charitable revenue amounted to £9,476, making the total income £37,162, against an expenditure of £41,659, thus showing a deficiency of about £4,500. The London Hospital had an assured income of £27,549, almost the same as Guy's, made up as follows: Invested property, £20,188; nursing institution, £2,258; legacies, £4,600; sundries, £503. The charitable revenue amounted to £28,633, making the total income £56,182, against an expenditure of £64,975, showing a deficiency of about £8,800. The London also received special legacies amounting to £13,739, which enabled them to pay their way. In making the awards the Distribution Committee first worked out the arithmetical basis—that is, the actual figures upon which each institution could claim a grant. This arithmetical basis in the case of the London Hospital worked out at £36,596. In the case of Guy's Hospital it worked out at £6,500. The unit of distribution based upon the amount raised by the Fund yielded this year 2s. 9d. in the pound. In addition to the arithmetical basis, the Distribution Committee add something according to the merits and needs of each institution. In the case of Guy's Hospital they made under this head a larger grant, viz., 7½ per cent. against 2½ per cent. given to the London Hospital, than to any other institution participating, with one exception—the Westminster. In other words, out of the moneys within their discretion the Distribution Committee gave 200 per cent. more to Guy's Hospital than they gave to the London Hospital. Turning to the merits and needs, Mr. Burdett pointed out that the cost of management at one of the institutions mentioned by Mr. Acland was three times as great as it was at another. Further, that whereas the London Hospital spent upwards of 50 per cent. more upon relief in 1894 than Guy's had, the London relieved 3,000 more in-patients and 72,000 more out-patients than Guy's Hospital. Surely the difference in the cost of management, which was 100 per cent. more at Guy's than at the London, and the difference in the amount of work done, in addition to the fact that the London Hospital had to raise £40,000 a year from voluntary sources, were adequate reasons to account for the difference in the amount of the grant received by the London Hospital as compared with that made to Guy's, having regard to the figures given above. If St. Thomas's Hospital wanted a grant, they would no doubt make application to the Hospital Sunday Fund in the same way as they had applied to the Hospital Saturday Fund.

Mr. Alfred Cohen supported the resolution.

The Chairman read the following passage from the letter of Sir Sydney Waterlow: "I also learn that Mr. Acland intends to submit a resolution for an alteration in the rules of the constitution in order to enable the Council to award a larger sum to Guy's and St. Thomas's Hospitals than can be awarded under the existing rules. I hope that the meeting will not hurriedly sanction an alteration in the laws of the constitution to give advantage to any one hospital over the others, and certainly not without asking the General Purposes and Distribution Committees to report upon the precise form in which it is suggested that the laws shall be altered. It should be urged upon the meeting that the present laws, although slightly modified since they were first framed, have worked well for many years past, giving general satisfaction to the institutions receiving a share of the money contributed on Hospital Sunday, and this is perhaps the best evidence of their fairness and justice. It must be remembered that, in comparison with other hospitals, Guy's and St. Thomas's have the advantage of a very large income from invested property as compared with their income from annual contributions, donations, &c. It is this fact which so largely governs the basis upon which the award to Guy's Hospital was based by the Distribution Committee last year. St. Thomas's Hospital has hitherto made no application for a share of the Hospital Sunday Fund. Should a claim be made in 1896 it will doubtless receive the most careful consideration of the committee. As vice-president of the Fund, and chairman of the Committee of Distribution ever since its formation, I naturally take the greatest interest in the work, and should view with great regret any change that would be likely injuriously to affect its hitherto harmonious working."

At the conclusion of the discussion the Chairman put the motion to the meeting, and it was carried by five votes to three, as, owing to the protracted character of the proceedings, the majority of the Council had left before the division.

A vote of thanks was accorded to the chairman for presiding, and the meeting then separated.

